# Toward a National Information Model for Medication Orders in Sweden

**DOI:** 10.1055/a-2546-4092

**Published:** 2025-04-21

**Authors:** Sofie Holmeland, Tobias Blomberg, Andreas Mårtensson, Sabine Koch

**Affiliations:** 1 Health Informatics Centre, Department of Learning, Informatics, Management and Ethics, Karolinska Institutet, Stockholm, Sweden; 2 Inera AB, Stockholm, Sweden

**Keywords:** health information interoperability, health information exchange, electronic health records, medical informatics, medication systems, data curation

## Abstract

**Background:**

Semantic interoperability among health information systems (HISs), in particular electronic health records (EHRs), is crucial for informed healthcare decisions and access to vital health data by the patient. However, inconsistent medication information and limited health data exchange contribute to medication errors worldwide. Although Sweden offers various solutions for health information exchange, there is a limitation in the exchange of medication orders and a lack of understanding the structure of medication orders among EHRs, highlighting the need for further exploration of the structure of medication orders.

**Objectives:**

This study aims to develop a common information model of medication orders for EHRs to be used in the Swedish context.

**Methods:**

An explorative qualitative design study was conducted. Documents and reference models of how medication orders are structured were collected, and semi-structured interviews were conducted with five purposefully selected participants with insight into how medication orders are structured in EHRs in Sweden. Data were analyzed using information needs analysis, information structure analysis, and code systems, classifications, and terminology analysis.

**Results:**

The following information areas were identified for a medication order: medication, medication indication, way of administration, medication order details, and dosage. These information areas were conceptualized into a Unified Modeling Language Class Diagram information model with defined classes, attributes, and data types. The resulting information model provides a representation of how medication orders are depicted in EHRs in Sweden and is aligned with existing national information models such as the National Medication List, while still providing additional information related to medication order details.

**Conclusion:**

The developed information model could potentially provide a national standardized model for medication orders, contributing to enhanced semantic interoperability and improving data exchange across various HISs. This could enhance data consistency, reducing the risk of medication errors and thereby improving patient safety.

## Introduction

Medication errors are a significant risk to patient safety worldwide. They can result in serious health complications, reduced quality of life, increased healthcare costs, and account for a significant number of deaths. In the United States, medication errors are ranked as the third leading cause of death. 1 In Sweden, around 1,400 patients die each year due to medication errors, and a total of 110,000 patients are in some way affected by medication errors. 2 Access to updated and correct medication information is an essential component to avoid medication errors. However, healthcare professionals face challenges when it comes to finding unified and correct information about a patient's medications as medication information is stored in decentralized and fragmented health information systems (HIS). Inconsistency of the information stored in different HIS and lack of interoperability lead to an elevated risk of medical errors. 1 A Swedish study revealed that only 55% of the information in medication lists from EHR systems and pharmacy systems align, emphasizing the need for improved data consistency. 3 Ideally, medication lists should be provided at a national level and give a correct overview of each patient's medication intake, irrespective of who ordered or prescribed the medication.

### National Medication Lists

**Fig. 1:**
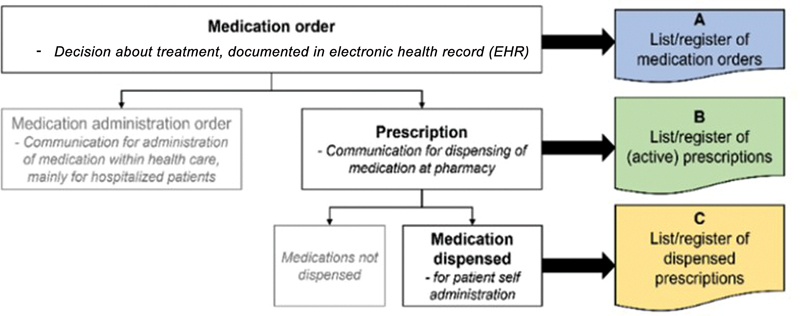
Flowchart of different types of National Medication Lists (NLL) and their origin. 8 NLL is based on the list of active prescriptions (B). This figure is taken from an article by Hammar et al published online with Open Access by IOS Press and distributed under the terms of the Creative Commons Attribution Non-Commercial License 4.0 (CC BY-NC 4.0). 8

There are initiatives to implement national medication lists in several countries, such as in the Nordic countries, 4 5 6 or in Austria. 7 In Austria, apart from prescribed and dispensed medication, over-the-counter (OTC) drugs are included in the medication list. Denmark and Norway base their medication list on medication orders, while Sweden and Finland base it on prescriptions. 8 The difference between the context of these medication lists is illustrated in Fig. 1 , where the Swedish medication list is based on list B, the list of prescriptions. 8

### Swedish eHealth Architecture

Sweden has taken several initiatives to address interoperability challenges between HISs over the years by developing a Swedish eHealth architecture consisting of different components. 9

#### National Reference Architecture

The National Reference Architecture functions as an installed base for the eHealth architecture. It defines architecture principles that cover the governing of eHealth projects, national functional scope, information security, loose coupling, local evolution within the national ecosystem, and federation. 9

#### Health Information Exchange Platform

**Fig. 2:**
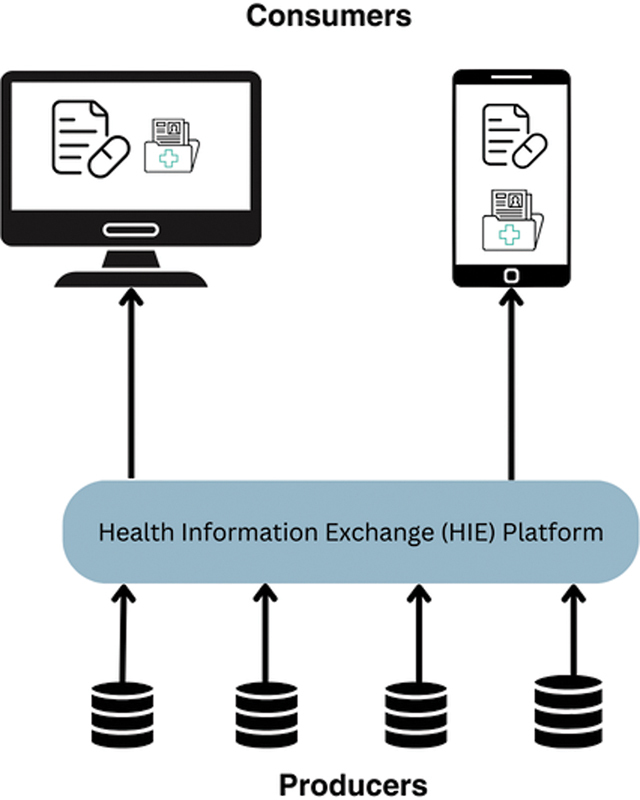
The health information exchange platform.

Healthcare in Sweden is decentralized to regions and provided by both public and private healthcare providers, leading to a variety of different HISs. 10 The Health Information Exchange (HIE) platform enables semantic and technical interoperability through service-oriented integration between these decentralized HISs. 9 Information is stored in one HIS (producer), and other HISs (consumers) can request this information through the HIE platform. Each HIS can be both a consumer and producer, depending on the specific information exchange scenario. 11 The communication between producers and consumers through the HIE platform is depicted in Fig. 2 .

#### National Service Contracts

Communication is facilitated through service contracts that define subsets of information to be exchanged for a specific process or function, such as medication history. 9

Get Medication History (GMH) is the service contract that defines the medication information shared through the HIE platform. 12 Compliance with GMH is voluntary for producers. Currently, 15 out of 21 regions, along with a few private healthcare providers, produce information according to GMH. 13 Moreover, it remains unclear which parts of the GMH service contract are available from the producers. 14

#### National eHealth Services

Sweden has several national eHealth services that use HIE for information exchange. The two major ones are the National Patient Summary (NPÖ) (Swedish: Nationell Patientöversikt) that provides a cohesive overview of patient information by compiling it from different EHRs. 15 This approach enables authorized healthcare professionals to access information recorded in different EHRs, provided that patients gave their consent. 16 17

The second service is “1177 Journal” which is accessible to Swedish inhabitants through the patient portal “1177.” “1177 Journal” gives patients access to the health records provided by their healthcare providers. 18 By centralizing patients' information in “1177 Journal,” patients gain access to their health record regardless of how many healthcare providers they have visited or the specific EHRs those healthcare providers use. However, patients' interactions with “1177 Journal” differ based on the healthcare provider's region or whether it is a private institution or not, as the healthcare provider determines the extent of available information. 19

##### National Medication Services

The Swedish National Medication List (NLL) (Swedish: Nationella Läkemedelslistan) aims to facilitate the interoperable exchange of medication information. 16 NLL is suggested to provide consistent and accurate medication information for a patient, ensuring patient safety by reducing medication discrepancies that pose a risk of causing medication-related issues. 8 20 When a decision about a medication treatment is initialized, it is registered in the EHR as a medication order. Transferring a medication order to a pharmacy is referred to as a prescription. It is essential to distinguish between these concepts since some medication orders do not necessarily become prescriptions, e.g., hospital internal/administered medications. When a prescription is generated, it is recorded and stored in databases overseen by the Swedish eHealth Agency, allowing pharmacy systems to access and retrieve the information. 21 The implementation of NLL in Sweden has been initiated by a law that promises to provide the exact same prescription information for the patient, healthcare, and pharmacies. 16 22 Integrating with the Swedish NLL is mandatory for all HISs that handle prescriptions or dispensing. 20 Due to various delays, the deadline for integration has been postponed several times, and the present deadline for mandatory integration is December 2025. 21 23 The information exchange in the Swedish NLL is based on the standard Health Level 7 Fast Healthcare Interoperability Resources (HL7 FHIR). 24

Patients can access their medication list of prescriptions and medication orders via the “1177 Journal” or access the medication list of their prescriptions through the Medication Check (Swedish: Läkemedelskollen). 19 25 Medication Check is a service provided by the eHealth Agency. The eHealth Agency further provides an overview of a patient's prescriptions intended for healthcare professionals, called the Prescription Check (Swedish: Förskrivningskollen). 21 This service is intended as a backup system before NLL is in full service, and the recommendation from the eHealth Agency is the primary source of a patient medication list to be from the EHR or NPÖ. 21 26 This is also a cause of concern, considering that not all healthcare providers produce the medication list through the service contract GMH, therefore not presenting it in NPÖ.

### Motivation of the Study

**Fig. 3:**
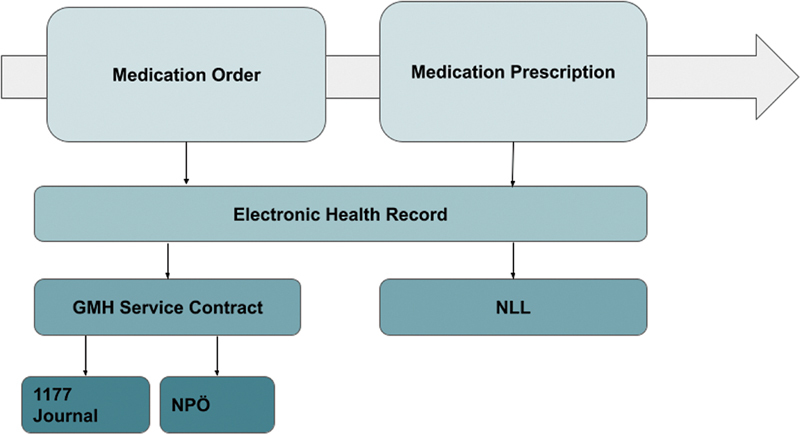
Information flow of medication order and medication prescription. GMH, Get Medication History; NLL, National Medication List; NPÖ, National Patient Summary.

In Sweden, despite the implementation of various initiatives, including the HIE platform, achieving interoperability for medication information remains a challenge. Since the NLL medication information is based on prescriptions and not medication orders EHRs have difficulties fully integrating with the NLL. 21 Further, only 15 out of 21 regions in Sweden share their EHR medication list through the HIE platform in accordance with the service contract GMH. 13 Consequently, a significant amount of medication information remains inaccessible to patients and healthcare professionals. Given that NLL already covers how prescriptions are modeled, there is a need for a new GMH service contract specifically focused on displaying the structure of medication orders in Sweden. The information flow of patients' medication lists in Sweden is depicted in Fig. 3 .

Although previous studies have addressed information modeling of prescriptions and EHRs, none has extensively explored the structure of medication orders in EHRs. 27 28 The information and its structure for medication orders in EHRs in Sweden remain ambiguous, indicating a need for further exploration.

The aim of this study is thus to develop a common information model for medication orders in EHRs in Sweden by identifying information elements that conceptualize medication orders. The information model is expected to provide deeper insight into how to enhance semantic interoperability for medication orders in Sweden. These insights can be utilized to create HIE solutions for medication orders, thereby enhancing patient safety.

## Methods

This study is a qualitative exploratory design study conducted in Sweden. Document analysis of European and national guidelines, reports, and existing information models regarding the medication domain was combined with five expert interviews. The design artifact resulting from this study is the developed information model. 29

### Data Collection

Key documents and reference information models were provided by the European eHealth network, the Swedish National Board of Health and Welfare, the Swedish eHealth Agency, and Inera AB (a company owned by the Swedish Association of Local Authorities and Regions, providing the national eHealth infrastructure). The list of collected documents is presented and described in Table 1 .

**Table 1:** Documents and reference models

Document	Provider	Description
** Guidelines on Patient Summary 30**	The eHealth network	The document is provided by a voluntary network set up by Directive 2011/24/EU. The purpose of the document is to provide a guideline of how health data for a patient summary should be structured to be shared both cross-border and nationally.
** Concept Model for Medication Ordering and Management within Healthcare 31**	The Swedish National Board of Health and Welfare	The concept model's purpose is to describe the concepts necessary for understanding the structured information of medication orders within healthcare. The model also gives an overview of the relationships between these concepts.
** Information Model for National Medication List 32**	The Swedish eHealth Agency	This information model provides a conceptual overview of how the information in the National Medication List is structured and related to each other.
** Medication Order and Management in Healthcare 33**	The Swedish National Board of Health and Welfare	The objective of this document is to offer direction on the implementation of the regulation and general advice (HSLF-FS 2017:37) within healthcare environments. HSLF-FS 2017:37 outlines the required information for medication orders, and this document offers guidance on how to integrate these guidelines into healthcare practices.
** Investigation on How the National Medication List Impacts on Ineras' Services 34**	Inera AB	This investigation provides results of how the National Medication List will influence different services provided at Inera. One of the outcomes includes an analysis of how the structure of the service contract “GetMedicationHistory” will be affected, along with proposed solutions.
** National Source for Medication Indication 2023 35**	The Swedish National Board of Health and Welfare	The National Source for Medication Indication (NKOO) (Swedish: Nationell Källa för Ordinations Orsak) is a code system, and this document presents its purpose, structure, and further development.
** Mapping of Datasets of National Interest in the Health Data Field—Final Report 36**	The Swedish National Board of Health and Welfare	This document provides the results from investigations regarding the national need for health data in different domains, where the results present the challenges and suggested solutions for data structures in hospital-supplied medication.
** Common Terms, Concepts, and Information Structure within the Medication Domain 37**	The Swedish National Board of Health and Welfare	Results from an investigation on how terms related to medication order and prescription shall be structured are presented in this document. These results also work as a contribution to the Swedish Social Welfare Board's concept model. 31
** Hospital-Supplied Medications in Epidemiological Studies 38**	Swedish Medical Products Agency	Hospital-supplied medications and how they are handled in Swedish health information system (HIS) have been investigated, and the results are presented in this document.

Semi-structured interviews in Swedish were conducted with five purposefully selected participants ( Table 2 ) with insight and knowledge regarding the structure of medication orders in different EHR systems that are used in Sweden. Questions in the interview guide were based on the main research question of how medication orders are structured in EHRs in Sweden, followed progressively by detailed and associated questions about classes, attributes, and code systems ( Appendix 1 , available in the online version).

**Table 2:** Interview participants

Interview number	Background	Employer
**1**	Pharmacist	EHR company 1
**2**	Requirement analyst	EHR company 2
**3**	Pharmacist	Hospital 1
**4**	Product owner	EHR company 3
**5**	Product owner	EHR company 4

The participants were invited by e-mail with information letters describing the purpose of the interview, participants' rights, and how the interviews would be conducted. Each interview lasted about 1 hour. Consent was given verbally and recorded before the interview started. The interviews were recorded using the Zoom meeting software and transcribed into Word.

### Data Analysis

Data triangulation was used by combining the collected data from the documents, reference models, and interviews, which underwent data analysis. 39 Combining data sources is beneficial for finding unified evidence, ensuring a clearer understanding of the research problem, and avoiding potential biases from single sources. 39 40

#### VIA Method

**Fig. 4:**
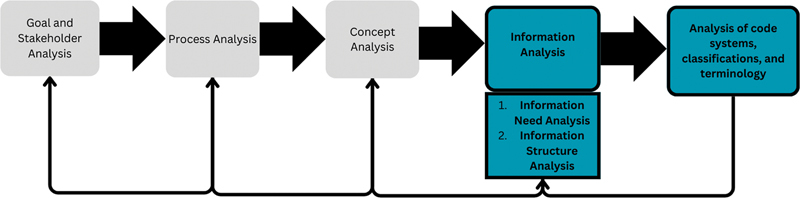
The Business and Information needs analysis (VIA) method flowchart. The highlighted steps were conducted during this study.

The Business and Information needs analysis (VIA) method (Swedish: Verksamhets- och Informationsbehovsanalys), a framework utilized by Region Stockholm for business and information needs analysis for healthcare organizations, served as the guiding methodological framework for the data analysis and development of the information model in this study. 41 The VIA method is an iterative method that involves five main steps and analyzes organizational needs from different angles, as illustrated in Fig. 4 . Each step has its aim and result to be followed up or preceded by another step. The steps can be utilized independently as standalone components, except step five, the analysis of the code system, classification, and terminology, that requires predefined attributes in an information model before it can be conducted. 41 The VIA method was deemed suitable for this study's purpose because it offers a methodological framework with flexibility and specific emphasis on information modeling in healthcare settings.

In this study, we used steps four and five from the VIA method, namely, *Information analysis* (including information needs analysis and information structure analysis) and *Analysis of code system, classifications, and terminology* .

##### Information Needs Analysis

The purpose of the information needs analysis is to itemize the information and produce results about what information goes in or out of an observed activity. Medication order was the observed activity in this study, and the analyzed information was the output from the medication order. The information volume for medication orders was extracted and analyzed from the documents and reference models. This was done by removing duplication of information and examining the significance of the information, i.e., analyzing the occurrence of words with similar meanings or words that have multiple meanings. Additionally, the information needs analysis included the interviews. Relevant information aligning with the information needed from the documents and reference models was identified. Furthermore, additional information needs related to medication orders, which were not present in the documents and reference models, were noted. This analysis followed abductive content analysis. Some information needs concepts were derived from the documents and were also found in the interviews, while others emerged solely from the interviews. 42 Relevant quotes from the interview transcripts were also marked to support the results of the information needs analysis.

##### Information Structure Analysis

The results of the information needs analysis, and the reference models worked as the basis for the information structure analysis in this study. From this data, an information model was developed. The information model was developed as a Class Diagram using Unified Model Language (UML). 43 The information model is neutral to any technical specifications and functions as a self-standing model which conceptualizes the defined information domain: medication order. 41 Identification of classes, associations, attributes, and multiplicity was done iteratively. In the iterations, the information model was reforged to align with the reference models and information needs analysis.

The step in the VIA method, referred to as “Analysis of code systems, classifications, and terminology,” was also incorporated into these iterative steps of information structure analysis. This step's purpose was to provide additional details and deepen the understanding of the attributes by adding another informational dimension to them. This step involved identifying which attributes in the information model could be linked to data types derived from different code systems, classifications, or terminologies, and to determine whether these linkages fulfilled the requirements for medication orders. The software tool Visual Paradigm 17.0 was used for developing the information model. 44

### Ethical Considerations

This study adhered to ethical guidelines for research involving human participants. Informed consent was obtained from all participants prior to their involvement in the study. The study was conducted in cooperation with Inera AB which at the time of conducting the study has been the affiliation of two of the authors (T.B. and A.M.). The generative artificial intelligence tools “Grammarly” and “ChatGPT” were used for grammatical, sentence structure, and translation support in this study. 45 46

## Results

### Information Needs Analysis

The information needs analysis identified several information areas related to a medication order, namely, medication, medication indication, administration, medication order details, and dosage ( Table 3 ).

**Table 3:** Results of the information needs analysis

Information area	Concepts	Occurrence in interviews
**Medication**	Medication product	5/5
	Medication name	5/5
	Medication form	5/5
	Strength	5/5
	Active substance	4/5
	Medication ID	4/5
	SIL	2/5
	NPL-id	3/5
	NPL-pack-id	1/5
	ATC	2/5
	Preparation instructions	2/5
	Package size	1/5
**Medication indication**	Treatment reason	5/5
	Modification reason	3/5
	NKOO	3/5
**Way of administration**	Administration route	5/5
	Administration method	4/5
	Place of administration	3/5
	Medical technical product	2/5
	Precision of place of administration	1/5
	Self-administered	2/5
	Administration instruction	2/5
**Medication order details**	Start and end date	5/5
	May be substituted	2/5
	Until further notice	2/5
	Follow-up	2/5
	Goal	1/5
**Dosage**	NLL dosage	2/5
	Immediate dosage	1/5
	Free text dosage	2/5
	Interval dosage	4/5
	One-time dosage	2/5
	Frequency of dosage	3/5
	Occasion of dosage	2/5
	Dosage amount	3/5
	prn	5/5
	Max. dosage	2/5
	Exceeding dosage	1/5
	Dosage speed	1/5

Abbreviations: ATC, Anatomical Therapeutic Chemical classification system; NKOO, National Code System for Medication Indication; NLL, National Medication List; NPL, National Product Register for Medications; prn, pro re nata; SIL, Swedish Information database of medical drugs.

#### Medication

The information structure of medication is a key concept that plays a central role in the medication order process. 33

Common information elements include the medication form, medication name, medication product, strength, and strength designation. 31 32 A medication name is a distinguishable name for a medication but does not include information about the medication's form or strength, which is necessary for the medication product. The medication product is a medication with a medication name, medication form, and strength. The strength is the amount of one active substance in the medication. In the NLL model, 32 this field is empty for medications with more than two active substances. The strength designation is a part of identifying and communicating about the medication product, and the strength designation can be used for up to three active substances.

The active substance is the component that gives a therapeutical effect or gives the possibility to set a diagnosis. An active substance can be referred to as a substance that is active on its own, or a compound, or the part of a compound that is active. 31 The attributes of active substances, strength, medication form, and brand name should be included in the European Patient Summary. 30 Some terminologies that should be used are Anatomical Therapeutic Chemical (ATC) classification system for the active substance, Unified Code for Units of Measurement (UCUM) or European Directorate for the Quality of Medicines & Healthcare (EDQM) for measurement of strength unit, and EDQM for medication form.

In Sweden, the National Product Register for Medications (Swedish: Nationellt produktregister för läkemedel (NPL))-id is used as a unique identifier of a medication product. 32 37 This includes license and extempore medication; however, some licenses or extempore medications do not have an assigned NPL-id. 38 Also, most products that lack an NPL-id are not, by definition, a medication, but they are still registered as such in the EHR medication module, for instance, enteral nutrition solutions, oxygen, or blood products. 38 Medications administered at hospitals are usually not registered by their NPL-id but rather by their medication name or active substance. Additionally, the ATC code is registered for hospital-administered medications, as mandated by the Swedish National Board of Health and Welfare. NPL-id can uniquely identify the medication and link it to its active substance, medication form, strength, and ATC code. However, the NPL-id identifies the original strength of a medication product and not the final strength before administration. This transition of strength occurs if a medication is prepared before administration, for example, powders that need to be mixed or medications that need to be diluted. 38

NPL-pack-id is another ID that uniquely identifies a certain medication article by its size and type. However, the NPL-pack-id is not relevant for medications administered at the hospital. 38 Thus, the structure of medication in the service contract GMH is not affected by the NLL's information model. 34

Results from the interviews confirmed the information content of medication. Medication ID is referred to as the NPL-id according to most interview participants, and many participants explained that it is also connected to the medication product, medication form, and strength. One participant mentioned that they register their medication ID with NPL-pack-id, although according to another participant, this is only necessary for a prescription to the pharmacy since NPL-pack-id tells which package type and size it is. When a medication is administered at the hospital, whichever package type and size is available from the medication cabinet will be given.

*“When you search for a medication, you often do not go by the ATC code but rather by the medication, form, strength, so the NPL-id.”*

Some participants mentioned that the ATC code for the medication product is registered, while one participant mentioned that this is not registered but is something that they plan to implement in the future. One participant mentioned that this was recorded for the medication order. Preparation instructions need to be provided for healthcare professionals who are managing the medication treatment. 33 This also aligns with the data from the interviews, where preparation instruction is mentioned as an attribute. The preparation instruction is addressed to the nurses who prepare the medication before administration and is relevant if the medication is a powder and needs to be mixed or diluted first. Interview participants stated that instructions could be recorded as free text or referred to an instruction document.

#### Medication Indication

Medication indication should be recorded for a medication order, 30 33 not only when a medication order is initiated but also when a medication order is modified or terminated. 33 The Swedish National Board of Health and Welfare has developed the National Code System for Medication Indication in Sweden, known as the NKOO (Swedish: Nationell Källa för Ordinationsorsak). All medication orders are advised to adhere to NKOO for their medication indications. 33 35 A selection of SNOMED CT terms in the NKOO facilitates the structured and nationally standardized registration of medication indications, which enhances communication between various HISs. 35 NKOO covers codes for both medication treatment reasons and medication modification reasons. The usage of SNOMED CT terms also aligns with recommendations for the European Patient Summary 30 regarding medication indication. NKOO establishes links between approved Swedish medications and their corresponding medication indications. It is compulsory to include the medication indication when the legislation (2018:1212) regarding NLL is in effect. The “Concept Model for Medication Ordering and Management within Healthcare” 31 incorporates medication indication as a part of the medication order structure, highlighting the utilization of NKOO for this purpose. There are two types of medication indication that can be registered: the initiation of the medication order and the adjustment of the medication order. 31 32 Further, one or several terms for the patient's understanding of the treatment purpose can be recorded in the medication order, intended for the patient as the recipient, and should be documented in the medication prescription. The “Concept Model for Medication Ordering and Management within Healthcare” 31 does not categorize treatment purpose under medication indication but rather as part of the medication treatment. The structure of the service contract GMH will be affected by the new structures of NLL and should, therefore, be reformed to align with these structures. 34 The NLL categorizes treatment purpose as part of the dosage instructions and not the medication indication. 32 The class medication indication in the NLL has an additional attribute called “description of other medication indication” in case none of the NKOO codes matches with the medication indication. 32

Data from the interviews confirm that medication indication is part of the medication order, with most EHRs already using treatment or modification codes from NKOO. Some EHRs, however, allow the use of free text to record medication indications even if it can be registered with NKOO. One reason being off-label medications, which means that the medication is not registered for a particular medication indication. Since NKOO only has connections between medications and their approved medication indications, it cannot be used for off-label medications. During one interview, the participant mentioned that off-label medications are very common among pediatric patients.

“ *In this NKOO (…) treatment reasons and modification reasons are included (...) And it will become mandatory with a national medication list that one has a treatment reason. Not all systems handle that registry.”*

#### Way of Administration

The way of administration needs to be recorded for a medication order. 33 It is referred to as the information of how a medication is administered to the patient's body. 33 Further, additional information can be recorded to ensure the safe handling of medications, such as the route of administration and medical technical products.

Common attributes include administration method, administration route, administration location, the precision of the administration location, and medical technical products used for the administration. 31 32 The administration method is the method of giving a medication, for example, injection. The administration route is the path for the medication to enter the patient's body, such as intravenous. The administration location is the bodily location of where the medication is given, for instance, the arm. This location could be further defined by the attribute precision of the administration location, such as right or left. Medical technical products used for administration should be mentioned, if necessary, as guidance for those who are going to administer the medication. 32 The structure way of administration in the service contract GMH is recommended to adapt to NLLs structure. 34 Data collected from EHRs in Sweden shows that the way of administration is an attribute that is recorded in many cases, 38 with only 2.3% of medication administrations missing the recording of “administration route.”

The document “Common Terms, Concepts, and Information Structure within the Medication Domain” 37 presents the results of an investigation, where a new code system with codes from a selection of SNOMED CT terms has been developed for way of administration. These terms have been mapped to EDQM terms that are used by the Swedish Medical Product Agency for regulatory medication information. This will also follow EU guidelines as the route of administration should be included with EDQM standard terms for patient medication history in the European Patient Summary. 30

Data from the interviews confirms that the way of administration is modeled in all the respective EHRs, although some only mention administration route. In one of the EHRs, administration route and administration method are linked. One participant mentioned that the way of administration is modeled according to Swedish Information database of medical drugs (SIL) framework. SIL's framework for the way of administration is the NLL information model. Some systems have the capability or are in the process of implementing SNOMED CT terms for the way of administration. However, there is an expressed doubt from one participant about whether all the attributes for the way of administration modeled in the NLL and concept model will be used in future EHRs.

There is also an expressed statement that there could be several ways of administration for medication and that this should be expressed for a medication order, and it is up to the one who administers it how it should be administered since it is perhaps necessary to adapt it to the patient.

*“We have customers who need to specify many different types of administration routes for a medication order (...) The person administering should be able to decide how to administer but within certain limits. You say this one can be given subcutaneously, intramuscularly, or as an injection. Or you may want to give it via PEG or different combinations so that it can be customized for the patient. The patient may not be able to swallow or something specific, or the patient is very thin... Then, you may have to give it intramuscularly instead of subcutaneously. Maybe there is no subcutaneous fat, and so on. It is important that it is open to adapt to the patient and the situation. There is a big difference if medication is to be administered at home or in intensive care, for example.”*

Self-administration is an attribute mentioned in two interviews as relevant to the way of administration, which can be true or false. The attribute tells if the patient is taking care of the administration; it is often used for medications administered in hospitals, according to the participants. The most common value for self-administration is false since most patients in hospitals get their medications administered by nurses.

Administration instruction is also something mentioned during the interviews, which is instructions on how the medication shall be administered to the patient, which is relevant for the nurses. It was mentioned by one participant that this is something that can be addressed to the patient and would be transferred to the prescription.

#### Medication Order Details

Treatment duration and follow-up are a central part of medication order. 31 33 The treatment duration is specified as either a time duration or as a condition, for instance, given once, until symptom-free, or until further notice. NLL information models do not have a specific treatment duration, although they have attributes for the first day of dosage and the last day of dosage, which combine into a dosage period. However, this refers to a specific prescription and not to the treatment duration of a medication order, which could have lasted longer or continued after the specific prescription was created. 32 A medication may (or may not) be substituted for an equivalent medication product, and this needs to be registered for a medication order. 33

Data from the interviews confirmed that the medication order has the attributes start date and end date, although the end date could be referred to as a condition, such as until further notice. The start date is the date when the medication treatment shall start, which might not be the same date when the medication order is created. One participant stated that this is a difference between a medication order and a prescription, where the prescription's start date is the same date when it was created.

*“You cannot really say that the prescription should be valid from... April 4th, but when you submit a prescription, it becomes valid from today... However, you can do that with a medication order. There, you can specify that the medication order should start from April 4th, and then it will not be available for administration at the hospital until that day, so to speak, for the nurse to administer. But you cannot do the same with a prescription because the patient must be able to pick it up. “*

The follow-up involves assessing the progress and effectiveness of a completed or ongoing medication treatment to determine any necessary further actions and shall be registered when the medication order is created following HSLF-FS 2017:37 guidelines. Follow-up can be registered as an exact date, a latest-before date, or that it will occur by the next doctor's appointment. 31 However, the NLL's information model 32 defines the follow-up as the latest-before date and refers to a prescription.

Follow-up is mentioned in three of the interviews, although it is noted that it is not always recorded in the EHR. One participant mentioned that the attribute follow-up is more frequently used for patients in hospitals. The follow-up is either expressed as free text or is structured with a date and time.

*“There should also be information on how medication treatment should be followed up and similar. And it is not always written either, but it should be included, and often it is written as free text in the medical records.”*

The medication order can have a goal for the patient, 37 such as being free from pain. The goal is recommended to be recorded but is not mandatory. One participant mentioned that the goal of medication order is recorded in their EHR.

#### Dosage

Dosage and administration occasion are information that should be registered where dosage should follow a structured format by HSLF-FS 2017:37, and administration occasion specifies the occasion or time of the administration. 33 Dosage has the attributes of dosage amount and dosage unit. The dosage amount refers to the specific amount of a certain medication with a certain strength and can be registered as a number, such as 2 or 20, or as an interval, such as 2–3. The dosage unit defines the measurement of dosage amount, which is distinguished by the medication form. For instance, the dosage unit can be milliliters (mL), milligrams (mg), international units (IU), tablets, capsules, sprays, or applications. 31 32 Periodicity is defined as a course characterized by the recurrence of the same condition at regular intervals, which specifies the time and condition for the administration occasion. The term in this context is also something that could be used for one-time dosage or given-as-needed dosage instructions. 31 If a medication is to be administered as needed, it needs to be documented as “pro re nata” (prn) and with the max dosage. 33 The NLL's information model has adapted the dosage to a structured format, with the possibility of free text options. The structured format involves different types of dosage structures, and some of them are modified from the “Concept Model for Medication Ordering and Management within Healthcare” attributes, dosage amount, and periodicity. 32 Dosage information should be structured according to the NLL's information model for the service contract GMH as the current GMH structure for dosage is complex, and it would be beneficial to adapt to the NLL structure since it is simpler and would make it easier for producers to connect to GMH. 34

Among the interview participants, there are differences in how the dosage for medication orders is documented. Some document the dosage through free text or short notifications, and some can use structured templates for dosage. Short notifications are abbreviations for dosage instructions used in EHRs in Sweden, and they are automatically transformed into whole dosage instructions.

Some participants described that the dosage is divided into different types of dosage and referred to them as structured dosages. One participant mentioned that these structured dosages are important for the flow in hospitals since when the medication is administered, there needs to be a dosage and a time reference. How these structured dosages are conceptualized differs, and some EHRs are in the process of adapting to the NLL's information model structured dosage while other systems are using their own way of structuring dosage. There is an expressed concern that the current dosage structure does not align with the NLL's structure and that will be one of the major challenges for many EHRs adapting to NLL. However, NLL's information model has adapted to this concern, too, by adding the ability to register the dosage with free text. This will solve the current situation for many EHRs, according to one participant.

*“The challenge for EHR now when they transition to NLL is because there is a distinct structuring for dosage in inpatient care in the medication order, and now NLL has devised a way to structure medication prescribing. And now these two systems must sync up so that what is a regular dosage in the existing systems' medication order aligns with how it is interpreted in NLL.”*

Regularly scheduled dosage and interval dosage are mentioned as a structured dosage form, which also aligns with the NLL's information model. The regularly scheduled dosage is given for medications that are given regularly or according to a schedule. The NLL's information model has two regularly scheduled dosages: frequency dosage and occasional dosage. 32

Common attributes that are mentioned during the interviews are dosage amount, dosage unit, periodicity, prn, max dosage for prn, and total max dosage per day. Another attribute is “first dose immediately,” which means that the medication needs to be administered as soon as possible, although it is not a mandatory field and perhaps more crucial if it is something acute, such as an EpiPen. If the dosage is not recommended or out of the normal scope, this needs to be noted in a comment. If a medication is given continuously, the dosage speed needs to be documented.

### Information Model

**Fig. 5:**
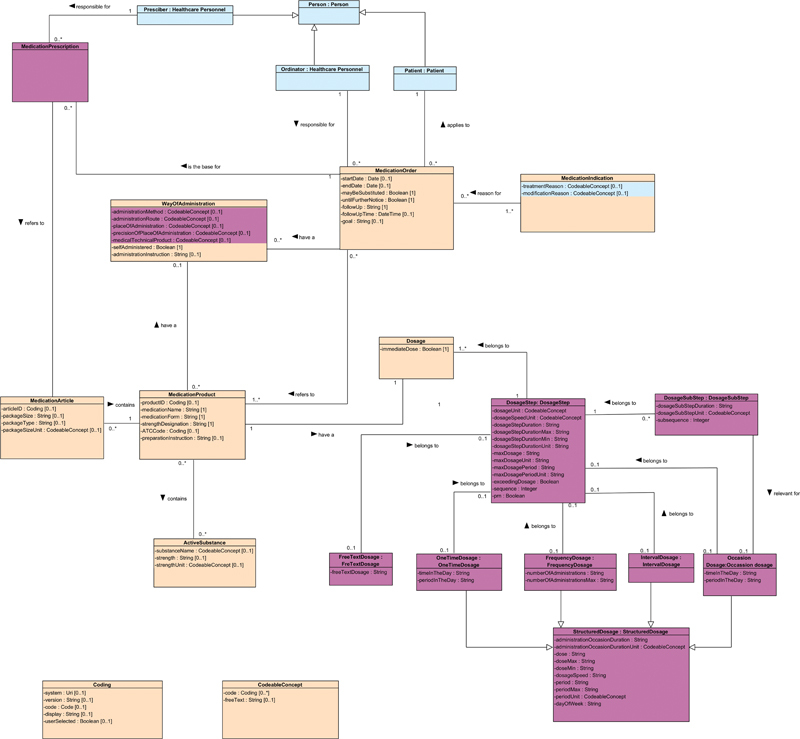
Medication order information model.

The developed information model depicting medication order is illustrated in Fig. 5 .

The classes “Person,” “Prescriber,” “Ordinator,” and “Patient” are based on reference models from the Swedish National Board of Health and Welfare, 47 and the class “MedicationPrescription” is based on the NLL's information model. 32 They are depicted in the information model to get an idea of how the medication order relates to these concepts and are not within the scope of this study's results. The default color of the information model is beige, where blue and purple colors are used to distinguish classes and attributes modeled according to reference models. Purple is depicted from the NLL's information model, the same color as the eHealth Agency signature color, but a lighter shade to make the readability easier. The light blue is the reference model's signature color from the “Concept Model for Medication Ordering and Management within Healthcare.” 31 32 47 Classes in the information model that are structured according to a reference model are referred to by the same class name with a colon. This approach aligns with how information models should be structured when referring to a reference model. 47

Detailed tables and descriptions of the different classes and their attributes are provided in Appendix 2 (available in the online version).

## Discussion

This study explored how information related to medication order is structured in Sweden by collecting data in the form of documents, reference models, and interviews. An information analysis resulted in the description of the information needs for medication order and an information model with some of its classes being based on the reference models from NLL 32 and the Swedish National Board of Health and Welfare. 47

### Findings

The information model provides an overview of how medication order is structured in EHRs in Sweden, which potentially can be used as a nationally standardized information model for Swedish healthcare.

#### Structural Relationship with NLL

Our information model for medication orders adds additional information compared to the NLL's information model, which focuses exclusively on medication prescriptions. Information areas for prescription and medication orders share overlapping structures, and we designed our information model to match the NLL's information model where applicable as it will be mandatory for all EHRs to conform to the NLL's information model.

A national standardized information model that encompasses both, medication orders and prescriptions, would enable information exchange between EHRs and even other HIS, such as pharmacy systems. However, a drawback could be the complexity of adaptation since there are more elements to consider. This might be one of the reasons why not all regions are connected to the service contract GMH, suggesting it would be beneficial to have a separate service contract solely for medication orders. Another consideration is that adaptation to NLL is mandatory for all EHRs, while connection to the service contract GMH is not. If medication orders were added to the NLL's information model, they would become mandatory as well, reducing the flexibility for the EHR developers.

### Comparison to Other Studies

Our information model exhibits a thorough structure for medication order compared to other studies. El-Sappagh et al propose an information model that could be applicable to EHRs. 27 When comparing our information model to theirs, it is evident that their model lacks a thorough analysis of the structure of a medication order, as the only relevant classes included are medication and prescription. Also, the classes have very few attributes, only medication ID for medication, prescription ID, and date/time for prescription. Similarly, a conference paper that focused on the structure of prescriptions for information exchange between EHRs and prescription systems did not deepen their analysis of the structure of medication order. 28 However, some of the attributes of the prescription class are present in our information model, such as medication ID, medication product name, strength, and unit of measurement for strength. Further, in the study by Manskow et al, 48 the dosage was free text and not in a well-defined structure, as in our medication information model. Comparing the results from these previous studies, this study excels in giving a comprehensive view of how medication order is structured, with a deeper analysis of the detailed elements.

### Strengths and Limitations

The choice of data analysis method, the VIA method, proved to be a major strength while conducting the information analysis for this study. The VIA method provided a framework that was easy to follow and proved most useful and well-adapted for information modeling in Sweden. The method has an adaptable approach, where not all steps need to be included. This flexibility proved particularly beneficial for this study's aim and objectives. The ease of use and flexibility suggest that this method could be adopted by other researchers, making it reproducible.

Furthermore, the reliability and validity of this study are strengthened by choosing a methodology that involves triangulation. 39 Triangulation of data collection through documents, reference models, and interviews made it possible to investigate medication orders from different perspectives, avoiding biases and ensuring accurate representation. Additionally, by interviewing individuals with insight into how EHRs are structured in Sweden, the collected data authentically reflects the actual configuration of these systems, enhancing the validity of the data.

Our information model has been developed without considering how it will be implemented, nor has it been implemented or evaluated yet.

### Implication of the Findings and Future Research

Our information model has the potential to serve as a foundation for a standardized model of how medication orders shall be structured for different technical implementations in Swedish healthcare. An important area of use for such a standard will be to provide requirements when developing integration specifications, such as service contracts of FHIR profiles, hence improving HIE between EHRs and other HISs by ensuring that the exchanged information remains relevant and valuable. If more EHRs are able to exchange this information, accessible patient information would increase for healthcare professionals, providing them with a comprehensive view of the patient's medication history. This improved access to data would set better preconditions for healthcare professionals to make informed decisions, potentially reducing the risk of medical errors and enhancing patient safety. Additionally, since the information model is neutral to technical implementation, such as HL7 FHIR or RIV-TA, it also makes it transferable for other scenarios or systems, independent of a particular system's technology.

This study focused on how medication order is structured in Sweden. Considering that Denmark and Norway have, or are on their way of establishing, NLLs based on medication orders, it would be of interest to see how these differ from this study's information model for medication order. 8 Future research could delve into comparing this information model with other information models, which could have the potential to further explore international information structure standards for medication orders.

## Conclusion

In this study, an information model has been successfully developed that presents a comprehensive overview of how medication orders are structured in current EHRs in Sweden.

Some information structures in the model align with the Swedish National Medication List (NLL) while still providing additional information related to medication order details. This could perhaps be utilized in a shared model that encompasses both prescriptions and medication orders to provide a holistic view of medication information. Compared to previous studies, our information model provides a deeper insight and representation of how medication orders are depicted in EHRs.

The developed information model could potentially provide a national standardized model for medication orders, contributing to semantic interoperability and improving data exchange across various HISs. This could enhance data consistency, reducing the risk of medication errors and thereby improving patient safety.
