# Supplementary material for: Toward a National Information Model for Medication Orders in Sweden
Source: Methods Inf Med. 2025 Apr 21;63(03-04):109–21. doi: 10.1055/a-2546-4092 (PMC12133328; doi:10.1055/a-2546-4092)
Supplement: Supplementary file 1 — Supplementary Material [file 10-1055-a-2546-4092-s24020008.pdf]

Information Specification

Classes and Attributes

MedicationOrder

The class “MedicationOrder” holds information about the medication treatment and its process. ►**Appendix Table A1** presents further descriptive information about the attributes.

Appendix Table A1 Description of the class “MedicationOrder”

Attribute	Mapping to a reference model	Description	Data type	Coding system
startDate	–	The date of when the medication treatment is going to be initialized. Must be recorded if the medication order is new.	Date [0..1]	–
endDate	–	The date of when the medication treatment is going to be stopped.	Date [0..1]	–
mayBeSubstituted	–	Informs if the medication may be substituted for an exchangeable medication in the medication order.	Boolean [1]	–
untilFurtherNotice	–	Presents if a medication is to be given until further notice, therefore not having an endDate.	Boolean [1]	–
followUp	–	Describes how the follow-up will be conducted.	String [1]	–
followUpDate	–	Presents the date and time if the follow-up meeting is set for a planned date.	DateTime [0..1]	–
goal	–	Presents a defined goal for the patient from the medication treatment, such as being free from pain.	String [0..1]	–

MedicationIndication

The class “MedicationIndication” holds information on the reason for the medication order. The “MedicationIndication” needs to be documented for the medication order, and either the treatment reason or modification needs to be recorded. ►**Appendix Table A2** presents further descriptive information about the attributes.

Appendix Table A2 Description of the class “MedicationIndication”

Attribute	Mapping to a reference model	Description	Data type	Coding system
treatmentReason	Concept Model for Medication Ordering and Management within Healthcare	Specifies the reason for treatment for the medication order. At least treatmentReason or modificationReason needs to be documented.	CodeableConcept [0..1]	Possibility to use a selection of SNOMED-CT codes from NKOO.
modificationReason	Concept Model for Medication Ordering and Management within Healthcare	Specifies the reason for modifying a medication order. At least treatmentReason or modificationReason needs to be documented.	CodeableConcept [0..1]	Possibility to use a selection of SNOMED-CT codes from NKOO.

 © 2025. The Author(s).

MedicationProduct

The class “MedicationProduct” holds information about a certain medication with specific properties. ►Appendix Table A3 presents further descriptive information about the attributes.

Appendix Table A3 Description of the class “MedicationProduct”

Attribute	Mapping to a reference model	Description	Data type	Coding system
productID	NLL information model. Medication product. NPL-id	Uniquely identifies the medication product.	Coding [0..1]	NPL-id codes from the NPL register provided by the Swedish Medical Products Agency
medicationName	NLL information model. Product. Product name	The distinguishable name for a medication product.	String [1]	–
medicationForm	NLL information model. Medication product. Medication form	Specify the form in which the medication is provided, such as tablet, capsule, or solution.	Codeable Concept [1]	Possibility to use EDQM standard terms or SNOMED-CT codes
strengthDesignation	NLL information model. Medication product. Strength designation	Provides information about the strength and unit of measurement of the whole medication product and can be used for up to three active substances, for example: “600mg/200mg/245mg”.	String [1]	–
ATCCode	–	Defines the ATC code for the medication product.	Coding [0..1]	The fifth level of ATC classification
preparationInstruction	–	Instructions if the medication needs to be prepared before the administration	String [0..1]	–

ActiveSubstance

The class “ActiveSubstance” informs about the component in the medication product that gives a therapeutical effect. ►Appendix Table A4 presents further descriptive information about the attributes.

Appendix Table A4 Description of the class “ActiveSubstance”

Attribute	Mapping to a reference model	Description	Data type	Coding system
substanceName	–	Name of the active substance	Codeable Concept [0..1]	Possibility to use SNOMED-CT codes
strength	NLL information model. Medication product. Strength	The amount of one active substance	String [0..1]	–
strengthUnit	NLL information model. Medication product. Strength unit	The unit of measurement regarding the amount of one active substance	Codeable Concept [0..1]	Possibility to use EDQM standard terms or UCUM codes

 © 2025. The Author(s).

MedicationArticle

The class "MedicationArticle" holds information about a specific type of medication product and its packaging. ►Appendix Table A5 presents further descriptive information about the attributes.

Appendix Table A5 Description of the class "MedicationArticle"

Attribute	Mapping to a reference model	Description	Data type	Coding system
articleID	NLLs information model. Medication article. NPL-pack-id	Uniquely identified the medication article.	Coding [0..1]	NPL-pack-id codes from the NPL register provided by the Swedish Medical Products Agency
packageSize	–	The size of the medication article, e.g.: 30 (tablets) or 3 × 10 (milliliter).	String [0..1]	–
packageType	NLLs information model. Medication article. Package type	The medication article type of packaging.	String [0..1]	–
packageSizeUnit	–	The unit of measurement for the size of the medication article, for example (30) tablets or (3 × 10) milliliters.	Codeable Concept [0..1]	Possibility to use UCUM codes

WayOfAdministration

The class "WayOfAdministration" holds information about how the medication is administered to the patient. ►Appendix Table A6 presents further descriptive information about the attributes.

Reference to NLL's information model: Way of Administration.

Appendix Table A6 Description of the class "WayOfAdministration"

Attribute	Mapping to a reference model	Description	Data type	Coding system
administration Method	NLLs information model. Way of administration. Administration method. Concept Model of Medication Ordering and Management within Healthcare: Administration Method.	The method used for administering the medication to the patient.	Codeable Concept [0..1]	Possibility to use SNOMED-CT codes
administration Route	NLL's information model. Way of Administration. Administration route. Concept Model of Medication Ordering and Management within Healthcare: Administration route	The route taken for administering the medication to the patient.	Codeable Concept [0..1]	Possibility to use SNOMED-CT codes
placeOf Administration	NLL's information model. Way of administration. Place of administration. Concept Model of Medication Ordering and Management within Healthcare: Place of administration	Where the administration takes place on the patient, such as the arm.	Codeable Concept [0..1]	Possibility to use SNOMED-CT codes

 © 2025. The Author(s).

Appendix Table A6 (*Continued*)

Attribute	Mapping to a reference model	Description	Data type	Coding system
precisionOfPlaceOf Administration	NLL's information model. Way of administration. Precision of place of administration. Concept Model of Medication Ordering and Management within Healthcare: Precision of place of administration	Defines a precise place of administration of the patient, such as right or left.	Codeable Concept [0..1]	Possibility to use SNOMED-CT codes
medicalTechnicalProduct	NLL's information model. Way of administration. Medical technical product for medication administration. Concept Model of Medication Ordering and Management within Healthcare: Medical technical product for medication administration	Identifies a medical technical product used for medication administration.	Codeable Concept [0..1]	Possibility to use SNOMED-CT codes
selfAdministered	–	Represents if the patient is self-administering or not.	Boolean [1]	
administrationInstruction	–	Detailed instructions are intended for the one who is administering the medication to the patient.	String [0..1]	

DosageStep

"DosageStep" is the class that holds information about the dosage and periodicity under a limited amount of time. ►**Appendix Table A7** presents further descriptive information about the attributes.

Referenced to NLL's information model: Dosage step.

Appendix Table A7 Description of the class "DosageStep"

Attribute	Mapping to a reference model	Description	Data type	Coding system
dosageUnit	NLL's information model. Dosage step. Dosage unit	The unit of measurement for the dose.	CodeableConcept	Possibility to use UCUM codes
dosageSpeedUnit	NLL's information model. Dosage step. Dosage speed unit	The unit of measurement for the dosage speed.	CodeableConcept	Possibility to use UCUM codes
dosageStepDuration	NLL's information model. Dosage step. Dosage step length	The duration of time for the dosage step.	String	–
dosageStepDurationMax	NLL's information model. Dosage step. Dosage step max	Defines the max time of the dosage step duration if it would be a time frame, such as 10–12 days.	String	–

(*Continued*)

 © 2025. The Author(s).

Appendix Table A7 (*Continued*)

Attribute	Mapping to a reference model	Description	Data type	Coding system
dosageStepDurationMin	NLL's information model. Dosage step. Dosage step min	Defines the minimum time of the dosage step duration if it would be a time frame, such as 10–12 days.	String	–
dosageStepDurationUnit	NLL's information model. Dosage step. Dosage step duration unit	Informs about the unit of measurement for dosage step duration min, such as days.	CodeableConcept	Possibility to use UCUM codes
maxDosage	NLL's information model. Dosage step. Max dosage	The max dosage for a certain time frame.	String	–
maxDosageUnit	NLL's information model. Dosage step. Max dosage unit	The unit of measurement for the max dosage.	String	–
maxDosagePeriod	NLL's information model. Dosage step. Max dosage period	The time frame for the max dosage, such as 1 day.	String	–
maxDosagePeriodUnit	NLL's information model. Dosage step. Max dosage period unit	The unit of measurement for the time frame of the max dosage, such as day.	String	–
exceedingDosage	NLL's information model. Dosage step. Obs. Exceeds recommended dosage	Indicates if the dosage exceeds the recommended dosage for the medication.	Boolean	–
sequence	NLL's information model. Dosage step. Sequence	A number that defines the order of the dosage step in relation to other possible dosage steps. Dosage steps that would occur during the same timeframe have the same sequence, while dosage steps that occur after each other have different sequence numbers.	Integer	–

DosageSubStep

The class "DosageSubStep" informs about possible sub-steps of a dosage step, only applicable to the class "OccasionDosage". ►**Appendix Table A8** presents further descriptive information about the attributes.

Reference to NLL's information model: Dosage sub step.

Appendix Table A8 Description of the class "DosageSubStep"

Attribute	Mapping to the reference model	Description	Data type	Coding system
dosageSubStepDuration	NLL's information model. Dosage sub step. Dosage sub step duration	The time duration of the sub-step in the dosage.	String	–
dosageSubStepDurationUnit	NLL's information model. Dosage sub step. Dosage sub step duration unit	The unit of measurement for the sub-step duration.	CodeableConcept	Possibility to use UCUM codes

 © 2025. The Author(s).

Appendix Table A8 (*Continued*)

Attribute	Mapping to the reference model	Description	Data type	Coding system
subsequence	NLL's information model. Dosage sub step. Subsequence	A number that defines the order of the dosage sub step in relation to other dosage sub-steps. Dosage sub-steps that would occur during the same timeframe have the same sub-sequence, while dosage sub-steps that occur after each other have different sub-sequence numbers.	Integer	–

StructuredDosage

The class "StructuredDosage" presents the dosage in a structured form. ►Appendix Table A9 presents further descriptive information about the attributes. For the attribute dayOfWeek, codes from the value set "Days of Week" provided by HL7 FHIR can be used.

Reference to NLL's information model: Structured dosage.

Appendix Table A9 Description of the class "StructuredDosage"

Attribute	Mapping to a reference model	Description	Data type	Coding system
administration OccasionDuration	NLL's information model. Structured dosage. Administration occasion duration	Specifies the time duration of the administration occasion.	String	–
administration OccasionDurationUnit	NLL's information model. Structured dosage. Administration occasion duration unit	The unit of measurement of the time duration of the administration occasion.	CodeableConcept	Possibility to use UCUM codes
Dose	NLL's information model. Structured dosage. Dose	Amount of a specific medication that is going to be administered to the patient.	String	–
doseMax	NLL's information model. Structured dosage. Dose max	If the dose is referred to as an interval, the dose max is the maximum dose for the interval.	String	–
doseMin	NLL's information model. Structured dosage. Dose min	If the dose is referred to as an interval, the dose min is the minimum dose for the interval.	String	–
dosageSpeed	NLL's information model. Structured dosage. Dosage speed	The dose per time unit is used together with the dosage speed unit.	String	–
Period	NLL's information model. Structured dosage. Period	The time frame of the dosage. For example: "2 tablets every day" period = 1 "2 tablets every other day" period = 2	String	–
periodMax	NLL's information model. Structured dosage. Period max	The max time frame used together with the period for an interval dosage. For example: "Every 6–8th hour" period max = 8	String	–

(*Continued*)

 © 2025. The Author(s).

Appendix Table A9 (*Continued*)

Attribute	Mapping to a reference model	Description	Data type	Coding system
periodUnit	NLL's information model. Structured dosage. Period unit	The unit of measurement for the period, such as days or hours.	CodeableConcept	Possibility to use UCUM codes
dayOfWeek	NLL's information model. Structured dosage. Day of week	Day of the week when the administration of the medication takes place.	CodeableConcept	Possibility to use HL7 FHIR codes for a day of the week

OneTimeDosage

The class "OneTimeDosage" informs about the dosage that shall be administered once and is a subclass of "Structured Dosage". Such as, 1 tablet is given once. ►**Appendix Table A10** presents further descriptive information about the attributes.

Reference to NLL's information model: One Time Dosage.

Appendix Table A10 Description of the class "OneTimeDosage"

Attribute	Mapping to a reference model	Description	Data type	Coding system
timeInTheDay	NLL's information model. One Time Dosage. Time in the day	The specific time when the medication is going to be administered.	String	–
periodInTheDay	NLL's information model. One Time Dosage. Period in the day	Informs which period during the day the medication is being administered, such as in the morning or the evening.	String	–

FrequencyDosage

The class "FrequencyDosage" informs about the same amount of dosage that is administered frequently. Such as one tablet once per day. ►**Appendix Table A11** presents further descriptive information about the attributes.

Reference to NLL's information model: Frequency Dosage.

Appendix Table A11 Description of the class "FrequencyDosage"

Attribute	Mapping to a reference model	Description	Data type	Coding system
numberOfAdministrations	NLL's information model. Frequency Dosage. Number of administrations	The number of repeated administrations during a period.	String	–
numberOf AdministrationsMax	NLL's information model. Frequency Dosage. Number of administrations max	The maximum number of repeated administrations during a period.	String	–

IntervalDosage

The class "IntervalDosage" informs about the same dosage that is administered between time intervals, such as one tablet every 8th hour. Even though the class do not have any attributes, it is a part of the NLL's information model. Therefore, it is modeled in the same way as for the medication order information model in this thesis.

Reference to NLL's information model: Interval Dosage.

 © 2025. The Author(s).

OccasionDosage

The class "OccasionDosage" informs about administered dosages bound to a specific time or period during the day and can be used for different amounts of dosages. ►Appendix Table A12 presents further descriptive information about the attributes.

Reference to NLL's information model: Occasion Dosage.

Appendix Table A12 Description of the class "OccasionDosage"

Attribute	Mapping to a reference model	Description	Data type	Coding system
timeInTheDay	NLL's information model. Occasion Dosage. Time in the day	A specific time for the dosage administration.	String	–
periodInTheDay	NLL's information model. Occasion Dosage. Period in the day	A specific period in the day for the dosage administration.	String	–

FreeTextDosage

The class "FreeTextDosage" provides information about the dosage with free text without the need for structured components. ►Appendix Table A13 presents further descriptive information about the attributes.

Reference to NLL's information model: Free text Dosage.

Appendix Table A13 Description of the class "FreeTextDosage"

Attribute	Mapping to a reference model	Description	Data type	Coding system
freeTextDosage	NLL's information model. Free text dosage. Free text dosage	The dosage is recorded as a free text without the need for structured components.	String	–

Dosage

The class "Dosage" holds information about the dosage amount during a certain period of time. ►Appendix Table A14 presents further descriptive information about the attributes.

Reference to NLLs information model: Dosage.

Appendix Table A14 Description of the class "Dosage"

Attribute	Mapping to a reference model	Description	Data type	Coding system
immediateDose		Informs if the medication needs to be administered as soon as possible and used for acute dosages.	Boolean [1]	–

Datatypes

Coding

"Coding" is used for attributes that can only be represented by a code or a term from a terminology or classification without the requirement of having the possibility of recording with free text. ►Appendix Table A15 presents further descriptive information about the datatype.

 © 2025. The Author(s).

Appendix Table A15 Description of the datatype “Coding”

Name	Data type	Description	Cardinality
system	Uri	Identifies the system of the terminology or classification.	[0..1]
version	String	Identifies the version of the system of the terminology or classification.	[0..1]
code	Code	The code identifies the concept from the terminology or classification system.	[0..1]
display	String	Identifies the representation of the concept.	[0..1]
userSelected	Boolean	Indicates if this coding was chosen directly by the user rather than assumed by the system the user works in.	[0..1]

CodeableConcept

“CodeableConcept” is used for attributes that could be documented with codes from a code system, but the possibility to record the attribute with free text is still needed. ►**Appendix Table A16** presents further descriptive information about the data type.

Appendix Table A16 Description of the data type “CodeableConcept”

Name	Data type	Description	Cardinality
code	Coding	The code is derived from the data type coding.	[0..*]
freeText	String	The concept is explained as free text.	[0..1]

 © 2025. The Author(s).
